# Indents

**Published:** 1913-06-15

**Authors:** 


### INDENTS.

☞Brief Articles of Technical or Scientific Value will receive notice and comment. Contributions invited.

*High-pressure Hypodermic Needle Packing.* Waxed floss silk wrapped around a shortened hypodermic needle used for a high-pressure anesthesia makes an absolutely tight joint.—W. H. Craft, *Dental Brief.*

*A Method of Giving Nitrous Oxid with the Addition of Ether in Minor Surgery.* Having witnessed the very painful recovery from nitrous oxid administered for the opening up of a whitlow, and having once had a similar experience himself, the author recommends the following method of administering nitrous oxid and ether. The mention of ether is sufficient to call up a vision of violent emesis; administered, however, as an addition to nitrous oxid in the following manner, it will be found exceptional that the patient becomes sick. The nitrous-oxid anesthesia is conducted in the usual way, but after the inhalation of the first bag of gas, which is, of course, refilled, the ether is turned on fairly quickly until the indicator stands at full ether. On the commencement of stertor, the anesthetic is withheld. This will produce an anesthesia of about twice the length of anesthesia by nitrous oxid alone. If a longer anesthesia than this is required, air is given from time to time. No to-and-fro breathing takes place at any time during the administration. The difference in the feelings of the patient on recovery from a short though painful operation under this anesthetic mixture as compared with anesthesia by nitrous oxid alone has to be experienced to be fully appreciated.—J. K. Pedley.

*Extirpating Pulps.* When it comes to the question of removing the pulp, the average dentist will do so, regardless of actual conditions, because of his fear that the pulp may subsequently give trouble. When we think of the pictures that have been thrown on the screen, showing tortuous root-canals from which pulps have been removed that could have been saved under proper treatment, it is a question whether we are conserving the patient's interest by so readily and indiscriminately, not to say ruthlessly, extirpating pulps as has been the practice in the past. I wish to be understood correctly. I am making no plea for the preservation of a pulp that is diseased to such an extent that it should be removed, but I am pleading for the conservation of pulps which are not sufficiently diseased to warrant removal.—Dr. Buckley, in *Cosmos, p. 410.*

*Importance of Integrity of Gums.* It is the writer's belief that a hard, elastic, pink gum is, beyond any shadow of doubt, the best guarantee of freedom from infection, both local and constitutional. The inner raw surface of the cuff or sleeve of the gum which surrounds the neck or root of a tooth whence the bony process has dissolved is a serious menace to the life and health of any individual—because it is easily possible that in an ordinary case of pyorrhea, involving perhaps the loss of an average depth of one-eighth of an inch of alveolar process the equivalent of two square inches of ulcerating raw surface is open to infection. The bone itself, being exceedingly cancellous, renders the spread of infection in its substance from such an ulcerating surface extremely easy, and the penetration of micro-organisms into the cancellous structures of the body of the bone is made certain by the force of occlusion —amounting to thousands of tons of pressure in a year—and this force being applied to the tooth causes the latter to act much like the plunger of a powerful syringe, injecting bacteria and the fluids of decomposition directly into the cancellous substance of the bone and the blood vessels and lymphatics of the surrounding tissues, thus producing deep inoculation by the bacteria that happen to be present in the pyorrhea pockets or apical abscess cavities.—Dr. T. B. Hartzell, in *April Cosmos, p. 371.*

*Infection from Dental Abscesses.* Dr. Moorehead, in the *Journal of the American Medical Association*, reported recently five cases of tubercular infection of the glands in close proximity to abscessed teeth, inferring that these glands became infected through the canals of abscessed teeth, and every dental and medical man knows that from such infected tubercular glands tubercle bacilli can be carried throughout the system, these bacilli attacking any part of the body that is below par. Dr. E. C. Rosenow, of Chicago, reported recently before the Chicago Dental Society six cases in which the patients had died from endocarditis, the post-mortem examinations showing that the endocarditis was the direct result of poisons absorbed by the blood-stream from pyorrheal pockets containing pus or abscesses associated with teeth. It is time then, that we stop blinking at these chronic abscesses in the mouths of patients, and if by therapeutics and surgery we cannot cure such conditions, let us extract such teeth, in order to avoid damage to the patient's systems.—Dr. Buckley, in *April Cosmos, p. 410.*

*Dental Diseases a National Menace.* Dental Disease is the most prevalent of all human ailments, and its very universality has obscured our vision too long to its real seriousness. What a difference it would make to the efficiency of the human race if all dental diseases could be prevented! And this really can be done by co-operation between the profession and the people. But of course this will not come about very soon. It is an exceedingly long journey to the goal of co-operation, and before this can be consummated it is necessary to educate the people as to what is involved in the question. But to go back of this and to work intelligently toward preventing these diseases we must first learn more of their etiology. We know but very little even yet about dental caries except that more than 90% of the people are affected by it. We know that an acid does the mischief and that microorganisms produce the acid, but we do not know why it is that in two mouths, both of which show the presence of these microorganisms, one will be immune and the other susceptible. Till we know this we cannot hope to prevent this disease.

What do we know about the causes lying back of erosion, or the real *modus operandi* of its progress? Almost nothing. What of pyorrhea alveolaris in its varying manifestations? Nothing except that it is rapidly becoming one of the most dreadful scourges which attack the structures surrounding the teeth, and cause their extensive loss. We are puttering and ever puttering in our efforts to check decay after it has begun, and to cure pyorrhea when it has already sapped the foundation of the teeth. We are using our ingenuity—an ingenuity not to be despised, either—in frantic efforts to undo the harm that has been done. And let me add in doing this we are giving the very best service of which we are capable with our present facilities. Lacking the knowledge of what causes these diseases we cannot be expected to do much toward preventing them. We have little need to blush for the character of our service to the people when all that we have learned relates only to repair and not to prevention.

But the time has come when a new obligation is being forced upon us. It is clearly our duty now, if it never was before, to institute a series of experiments with the aim of learning the causes of dental diseases and the surest means of preventing them. The question is, how shall this be done? The first requirement is money, the next requirement is money, and the next is more money. I do not mean by this that vast sums are needed to carry on this work but that it is folly for any investigator to start in without the assurance of financial support during an indefinite period. To do this movement justice there should be an endowment suitable in size to sustain a laboratory of research pe manently. To start it without adequate means to support it on a permanent basis is to waste so much energy. No man can do this work to advantage and earn his living practicing his profession. He must be relieved of financial worry to gain the best results, and this must be a permanent instead of a temporary relief.

Logically this work should be undertaken by the government, but practically this is not feasible at the present time. Our government is not yet educated up to it. The government has only gone far enough to look after the welfare of hogs and cattle, and sheep. It is absorbed with the problem of growing the best kinds of grain and fruits. It takes a vital interest in the health of domestic animals, and will aid a farmer most effectually in running down the causes of disease in his herds or flocks. This is done because these herds or flocks represent so much money and the government has learned the significant lesson that whatever is a saving to the individual is a saving to the community. It is perfectly proper and entirely commendable for the government to do these things. It is for the betterment of the state, and the nation and the family.

But the government has one very important lesson to learn, viz., that the health of the people is of greater importance than the health of its domestic animals, and that from a mere mercenary point of view, to say nothing of sentiment, it is a saving of dollars and cents to keep the people free from disease. Human health is the greatest asset any nation can have. It has been estimated by those who have studied the economic aspect of the case that the cost of disease each year among the people of this country alone runs into an enormous sum—up in the billions. In a recent number of the *Bulletin* issued by the Department of Health of Chicago the statement is made that "the sum of $1,500,000,000 is a low estimate of the annual economic loss from preventable deaths." The extravagance entailed in the high cost of living which we hear so much about today is as nothing compared with the extravagance of disease. It is the cruelest waste in this age of excessive wastefulness. If the government would spend one-half the sum which is wasted annually by disease in maintaining a department of health to teach and direct the people how to keep well, and then establish original research laboratories to seek out the causes and prevention of disease, it would be the most economical expenditure of money that could be devised.—C. N. Johnson, in *Review, p. 415.*

*A Call for Research Laboratories.* It is a splendid thing to go out into the poorer districts of the city and relieve suffering, thereby increasing the efficiency of those whom we serve, but it is infinitely a higher mission for us to find out the causes of these diseases which we are today combating, to the end that we may ultimately be able to prevent them. The curing of a disease, as I have already pointed out, is a costly thing, and it savors of that kind of extravagance which is the most woeful of all because it is unnecessary. Not that we are at present able to escape it, not that we are falling short of doing all in our power with our present knowledge, but that the time has come in our evolution to higher and better things when our energies should be directed toward the prevention instead of the cure of disease.

And the sooner we are permitted to establish laboratories of original research the sooner will this consummation so devoutly wished, be brought about. I would that some of our wealthy men—men who are seeking unselfishly and generously to benefit the world—could be made to see the significance of this work. If they could understand as we today are beginning to understand the real value to the human race of mouth hygiene there would be not only one but literally there would be dozens of them who would volunteer to finance laboratories to throw light on this important subject.—C. N. Johnson, in *Review, May 1913. p. 418.*

*The Specialist.* I believe in specializing in both dentistry and in medicine. It has been justly said that a great deal of the wisdom of a man in this twentieth century is shown in leaving things unknown, and there is a great deal of practical sense in leaving things undone. The day of the universal scholar has passed. "Life is short and art is long." The range of human knowledge is so great that no one brain can grapple with it, and he who would know one thing well must have courage to be ignorant of a thousand other things, however attractive or inviting. As with knowledge, so with work. The man who would get along, must single out his specialty and into that must pour the whole stream of his activity, all the energies of his hand and tongue, heart and brain. Broad culture may mean beautiful things to contemplate, but after all, it is the narrow edge man, the man of single and intense purpose who throws his soul against all things else, that accomplishes the hard work of the world. With the exception of a few great creative minds, the men whose names are historic have been identified with some great achievement upon which all their life force is spent. You think of Watt and instantly the steam engine is suggested: you think of Davy and the safety lamp that lights up the mine is suggested; of Harvey, and the bood courses more quickly through your veins; of Jenner, and you see disease stayed in its progress by the discovery of vaccination; of Morse, and the electric spark is seen darting from continent to continent; of Edison, and the electric light flashes before your eyes; of Marconi, and you see hundreds of human lives saved from sinking vessels.

I am aware that this concentration of oneness of aim is not always to be desired. There have been prodigies, geniuses, who have accomplished a great many things during an ordinary lifetime, and they have done these many things well. Cicero was a master of logic, of ethics, of natural philosophy, besides being versed in geometry and music and the other fine arts. Leonardo da Vinci was not only a great painter, but a mathematician, metaphysician, musician, poet, architect sculptor, chemist, anatomist, besides being well up in mechanics and natural history. The very rarity of such prodigies is what makes them prodigies. While one such man, who is fully equipped with these possibilities, may impart knowledge, there are thousands who have made a failure in life by dabbling in too many things. Most men are not unsuccessful if they have only known a few things. It is far better to be ignorant of many things and thus escape the calamity of being ignorant of everything. I do not mean, understand, that man should be a mere cog or pulley in the machinery of the world. He should be a man, first of all. He should prepare himself fully for the avocation which he follows in life, and there is no avocation in life for which any man feels himself fitted that he cannot serve with distinction and perhaps honor. All work is honorable, no matter what it is. The man who serves his fellow man, and does what he can, to lift the souls of men out of the darkness into God's effulgent light, has done great work. You men of the medical profession, who are ameliorating the sufferings of humanity, are doing a grand and noble work. And you gentlemen of the dental profession, who are saving human kind from hours of agony and are giving comfort to them throughout their whole lives, are doing a grand work. In that, all of us are trying to do our duty as best we can.—Dr. E. T. Darter, in *Dental Review, May 1913, p. 397.*

*Unclean Mouths a Menace to the Community.* I would impress upon you, then, that the condition of the mouth and of the teeth is not a matter of only local importance. It affects the individual's whole body, and not the mouth alone Its influence goes even farther than that. I believe honestly and earnestly that the time will come when the condition of a person's mouth will be taken as much into account by the municiple authorities as the condition of his morals is taken into account to some extent today. The time will come when no one will be allowed by the municipality to carry around an immoral mouth, as it were. This is only a question of the *mores,* of the custom of the time. At present the opinion prevails that the condition of a person's mouth is his own private affair, and if you went up to a man on the street today and told him that his mouth was a menace to the community, and he must either clean it or go to jail, he would probably knock you down or consign you to a place which is even hotter than Washington—if any such place there be. The time is coming when a dirty, immoral mouth will be considered as much a menace to the community as an infectious disease, and the person with such a mouth will be quarantined the same as the person who has smallpox. And that is the thought I want to leave with you—that the condition of a person's mouth is not only of importance to that person, but is a matter of moment to the community at large; that a dirty, uncared-for mouth is not only a source of disease to the individual, but also a menace to the health of every person who comes within a reasonable distance of him. When viewed in that way, it can readily be seen that the condition of your mouth is not only your business, but is my business, and everybody's business; it is the business of the community.—Dr. G. E. Hunt, in *April Cosmos, p. 429.*

*Method of Reinforcing Plaster Casts.* Many times we find that when our models are made they are very thin, too thin and weak to withstands the pressure necessary in closing the flask. We also find abutments breaking off the model in bridge work, etc. An easy, cheap, and sure method to overcome this difficulty is to get some brass wire gauze, to be found at all tin shops—this gauze is used for milk-strainers, etc. For reinforcing crowns used as abutments in bridge work, a piece about one-half inch long, and one-half of the size of a lead pencil is rolled up, and after dipping it into water it is gently worked into the crown, when pouring the investments in to get the model. If a lower impression has a very thin ridge, and the operator ready to make the model, a piece of the gauze is cut, about two inches wide and long enough to go from one end of the model to the other. Then the gauze is folded in the middle and as the plaster is being poured into the impression the smooth edge of the gauze, not the cut edge, is worked into the plaster, holding it for a moment until the plaster begins to set.—J. M. Prime, *Dental Brief.*

*Removing Enamel from Occlusal Surfaces of Teeth in Preparation for Crowns.* Two, three, or four knife-edged carborundum disks are mounted together, and grooves are cut as deep as the thickness of enamel to be removed. By keeping the disks moist, this can be done almost entirely without pain. With a chisel placed in the grooves, the entire occlusal surface can be chipped off.—H. A. Mares, *Dental Review.*

*The Gingival Wall.* Cavities are often cut with the gingival wall in the form of a curve connecting the buccal and lingual walls. This leaves a little triangular area of enamel at each angle that is more susceptible to caries than much of the enamel that has been cut away. It is the writer's belief that if all operators made no other change in their present cavity preparations than to square out these angles, the average life of our proximal fillings would be doubled. I make this statement because these areas are the most susceptible positions on the enamel remaining, as evidenced by the fact that recurrences of caries begin in these positions in the large majority of cases.

For purposes of retention and convenience in placing fillings or inlays, the box-like form of cavity, with the surrounding walls nearly parallel and all angles sharply cut, answers all requirements. Such cavities have the best mechanical forms for retention and for resisting the stress of mastication, and the fillings may be started and placed with greater ease and facility than in cavities of more rounded form.—Dr. A. D. Black in May *Cosmos.*

*Extension for Prevention.* It is certainly rational to apply our knowledge of pathology in cavity preparation; in fact, this knowledge should be our guide in determining the outline form of the cavity. If a cavity is presented which has not involved *on the surface* all of the enamel which our observation and records show to be clearly susceptible—enamel on which micro-organisms will grow and cause caries irrespectively of whether we place a filling or not—is it not very clearly our duty to include that susceptible enamel in the cavity outline? I believe practically all will agree with me in this statement, but we may differ in the application of these principles in practice.

To illustrate the application of this rule I will cite three different proximal cavities, with the treatment adapted for each. The first is a small area of superficial caries *which has not spread over the full area of susceptibility;* the caries has penetrated the enamel, and there is an area of dentin involved which is *less wide* than the involved surface of the enamel. The treatment requires that the remainder of the *susceptible area* of sound enamel be cut away so that the margins of the filling will be placed in immune areas. *That is extension for prevention.*

The second case is one of caries *which has spread over the full area of susceptibility* on the surface of the enamel, and the area of dentin involved is *less wide* than the involvement of the enamel. The treatment of this case requires that the cavity include all the affected enamel, which here comprises all of the susceptible area. It would not therefore be necessary to cut away any sound enamel; the outline of the cavity would, however, be exactly the same as in the first case. *Extension for prevention would be unnecessary, and would not apply in this case.*

The third case is one of caries *which has spread over the full area of susceptibility on the surface,* and the area of dentin involved is *considerably wider* than that of the enamel; considerable enamel, unaffected on its surface, has been undermined by lateral caries in the dentin. In this case, the caries in the dentin has undermined not only the enamel susceptible to caries on its surface, *but also the immune enamel* farther toward the buccal and lingual angles, possibly including the angles, or even beyond. The extent of caries in the dentin, therefore, controls the outline form of the cavity and caries it far beyond the position demanded by the rules of extension for prevention. *Extension for prevention* is therefore uncalled for in the treatment of caries of this type.

These three cases are cited to emphasize the fact that extension for prevention applies to but one type of cavities —those in which the surface caries has not extended far enough to include the entire area of susceptibility, and in which the caries in the dentin has not undermined the enamel of this area. Extension for prevention applies to no other cavity, because it involves only the cutting away of sound enamel within the area of susceptibility, and it is obvious that no sound enamel in this area could be cut away if the surface spread of caries, or the lateral spread in the dentin underneath, has rendered it unsound.—Dr. A. D. Black in *Cosmos*.

